# Solvothermal synthesis of soluble, surface modified anatase and transition metal doped anatase hybrid nanocrystals

**DOI:** 10.1039/d2na00640e

**Published:** 2022-11-11

**Authors:** A. S. Bathe, A. Sanz Arjona, A. Regan, C. Wallace, C. R. Nerney, N. O'Donoghue, J. M. Crosland, T. Simonian, R. I. Walton, P. W. Dunne

**Affiliations:** School of Chemistry, Trinity College Dublin, College Green Dublin 2 Ireland p.w.dunne@tcd.ie; CDT ACM, AMBER, Trinity College Dublin, College Green Dublin 2 Ireland; School of Chemistry, University of Warwick Gibbet Hill Coventry CV4 7AL UK

## Abstract

Titanium dioxide, or titania, is perhaps the most well-known and widely studied photocatalytic material, with myriad applications, due to a high degree of tunability achievable through the incorporation of dopants and control of phase composition and particle size. Many of the applications of titanium dioxide require particular forms, such as gels, coatings, or thin films, making the development of hybrid solution processable nanoparticles increasingly attractive. Here we report a simple solvothermal route to highly dispersible anatase phase titanium dioxide hybrid nanoparticles from amorphous titania. Solvothermal treatment of the amorphous titania in trifluoroacetic acid leads to the formation of anatase phase nanoparticles with a high degree of size control and near complete surface functionalisation. This renders the particles highly dispersible in simple organic solvents such as acetone. Dopant ions may be readily incorporated into the amorphous precursor by co-precipitation, with no adverse effect on subsequent crystallisation and surface modification.

## Abstract 2

Solvothermal treatment of amorphous titania in trifluoroacetic acid modifies the surface and crystallises the anatase phase, yielding dispersible hybrid nanocrystals.

## Graphical abstract

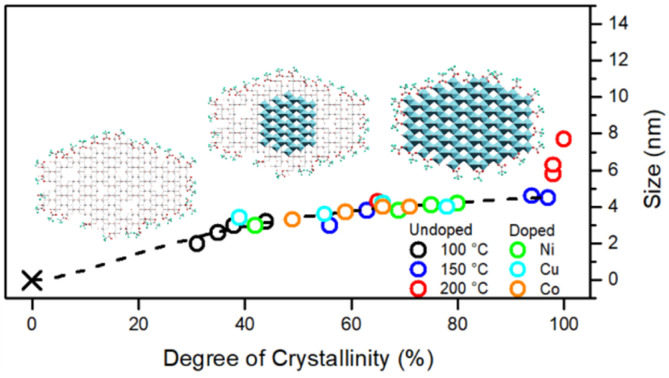

## Introduction

Metal oxides exhibit an array of properties which have made them integral to many applications, from the mundane, to the exotic. Among the oxides one of the most widely used and studied is titania, TiO_2_. Long exploited as a pigment for its brilliant whiteness, titania is also the prototypical photocatalyst. Since the seminal work of Fujishima and Honda in 1972 its photocatalytic behaviour has been well documented in applications ranging from water splitting and energy applications, to environmental remediation of pollutants, to air-fresheners, to biosensors.^1–7^ Titania commonly exhibits three crystalline polymorphs, anatase, rutile, and brookite, and several amorphous structures have been proposed.^8,9^ Of the polymorphic forms adopted by titania, the anatase phase has typically been identified as the most photocatalytically active (and is the major component of the commercial photocatalytic standard Degussa P25).^10–12^ Numerous strategies have been adopted to further improve its performance and efficiency. Chief among these is the incorporation of dopants to modify the electronic structure, whether that be by substitution of Ti^4+^ for an alternative metal ion, or the incorporation of heteroatoms such as carbon, nitrogen or fluorine.^13–16^ This can serve to decrease the band-gap, extend the absorption into the visible range, and prevent electron–hole recombination, each of which may serve to increase the efficacy of the photocatalyst.

While there are innumerable reports on the production of titania and doped titania nanoparticles,^17–19^ it is worth noting that many of its applications require a specific form, *i.e.*, supported particles, gels, coatings, or thin-films.^20,21^ As with most metal oxides, titania is a highly intractable material – once prepared it is not easily manipulated. For this reason, vapour deposition techniques are very often employed, particularly in the generation of coatings and thin films. While such techniques have clear advantages, they do suffer some significant drawbacks, including high capital costs and often the need for high vacuum.^22,23^ Additionally, the generation of doped materials by vapour techniques presents some difficult challenges in choice of precursors and control of phase separation. An increasingly popular alternative to vapour phase methods is the generation of solution processable metal oxide nanoparticles.^24–26^ This approach offers exceptional flexibility, both chemically – simple wet-chemical techniques permit the synthesis of a wide range of compositions – and physically, as dispersions of preformed colloidal nanocrystals may be used to coat flat substrates, irregularly shaped objects or may be used to generate supported catalysts or composites and gels.^27–32^ There has been a growing research effort in this field, such that dispersible nanocrystals of TiO_2_, SnO_2_, CeO_2_, ZnO, MnO, and others have been accessed by methods including non-aqueous sol–gel synthesis, solvothermal/hydrothermal synthesis, hot-injection, and thermolysis.^13,29,32–37^

Previously we have reported on the production of highly dispersible tin oxide and titanium oxide nanocrystals by post-synthetic modification of hydrous oxide precursors.^38,39^ This was achieved by the precipitation of the hydrous oxide and subsequent surface modification with the simple short chain carboxylic acids acetic acid and trifluoroacetic acid under reflux conditions. In the case of titania, it was found that the poorly crystalline hydrous titania partially transformed to nanocrystalline anatase under these conditions. Here we extend this approach to the generation of highly dispersible transition metal doped anatase nanocrystals, whereby solvothermal treatment allows the controlled crystallisation of the anatase phase while simultaneously modifying the nanocrystal surface.

## Experimental

### Synthesis

In a typical synthesis 6.85 mL (6.25 × 10^−2^ moles) of titanium tetrachloride (99.9%, Fluorochem, Ltd) was added slowly to a large excess of distilled water (200 mL) under constant stirring. *N.B.*, this is an extremely vigorous reaction, liberating large quantities of HCl vapour, and appropriate care should be taken. Hydrous titania was precipitated from this solution by the slow addition of 10 M sodium hydroxide with constant monitoring up to a pH of 6. The white hydrous titania product was isolated and washed by repeated centrifugation until free of chloride, as determined by the silver nitrate test. The obtained solid was allowed to dry under ambient conditions to a damp but friable state.

1 g of the hydrous precursor was stirred in 10 mL of trifluoroacetic acid (TFA) for 5 minutes in 23 mL Teflon lined stainless steel autoclaves. The autoclaves were then placed in a pre-heated electric oven for solvothermal treatment at the desired temperatures of 100, 150, and 200 °C for 3, 6, 17 and 24 hours. After this time the autoclaves were removed from the oven and allowed to cool under ambient conditions. Excess TFA was removed from the obtained dispersions by rotary evaporation, yielding fine, dry powders.

For comparison the same hydrous precursor was subjected to hydrothermal treatment by heating 1 g of the material in 10 mL of distilled water at 100, 150, and 200 °C for 3, 6, 17 and 24 hours in Teflon lined stainless steel autoclaves. The solid products obtained by the hydrothermal route were isolated by filtration.

Nickel, copper, and cobalt doped analogues were prepared in the same manner; however, the titanium tetrachloride was added to solutions containing appropriate amounts of NiCl_2_·6H_2_O, CuCl_2_·6H_2_O, or CoCl_2_·6H_2_O (Fisher Scientific, Ltd), respectively, to yield products at 5 mol% doping. These dopants and dopant levels were chosen to provide a proof-of-principle that this approach would be viable for the generation of dispersible doped titania. The doped hydrous precursors were treated hydrothermally and solvothermally in TFA at 150 °C for 3, 6, 17, and 24 hours.

All solid products obtained by solvothermal treatment were found to be highly dispersible in acetone without sonication or other treatments, as per our previous work.^38,39^

### Characterisation

X-ray diffraction (XRD) patterns were recorded on a Bruker D2 benchtop diffractometer using Cu Kα radiation from 5° to 70° 2*θ* with step-size 0.01° at 1.5 s per step using a zero-background Si sample holder. Data fitting was performed using the XFit and Fityk software packages.^40,41^ Infrared spectra were recorded on a PerkinElmer Spectrum 1 ATR-FTIR instrument from 4000 to 650 cm^−1^. Thermogravimetric analysis and differential scanning calorimetry (TGA/DSC) were performed using a Mettler–Toledo TGA/DSC1 under an air atmosphere with a heating rate of 5 °C min^−1^. Transmission electron microscopy (TEM) images were obtained on a JEOL 2100 operating at an accelerating voltage of 200 kV and a FEI Titan 80–300 kV S/TEM operating at 300 kV. Energy-dispersive X-ray analysis (EDX) was carried out using a Zeiss ULTRA plus Cryo SEM, equipped with an 80 mm^2^ Oxford Instruments EDX detector, with an energy resolution of 129 eV.

## Results and discussion

**Fig. 1:**
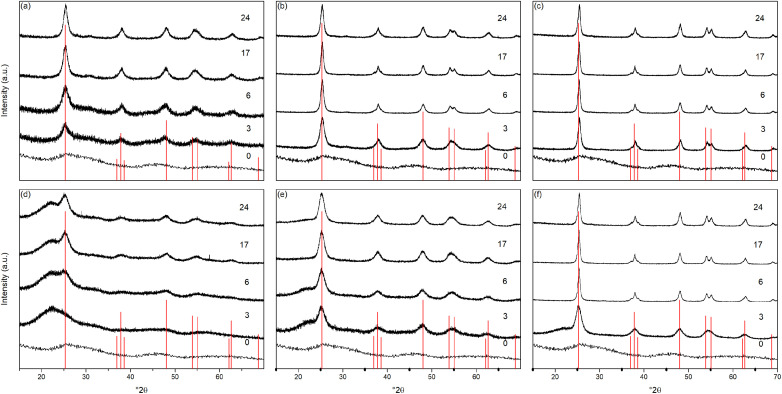
X-ray diffraction patterns of undoped titania treated hydrothermally for the indicated times at (a) 100 °C, (b) 150 °C, and (c) 200 °C, and solvothermally in trifluoroacetic acid for the indicated times at (d) 100 °C, (e) 150 °C, and (f) 200 °C. Data have been normalised and offset for clarity.

The X-ray powder diffraction patterns of the undoped samples treated hydrothermally are shown in Fig. 1(a)–(c). The hydrous oxide precursor prepared by precipitation at pH 6 (*i.e.*, time = 0) is very poorly crystalline, with only very broad features observed, with diffuse scattering centred around 29° 2*θ* and additional shallow features at lower *d*-spacings, likely attributable to hydrogen titanate type species.^42–46^

Upon hydrothermal treatment at 100 °C the amorphous hydrous titania crystallises into the anatase phase with some residual amorphous features. Increasing the hydrothermal treatment time leads to growth of the anatase phase and a decrease of the amorphous features, which resolve to a very weak but distinct diffraction peak at 30° 2*θ*, corresponding to the (211) peak of brookite. The degree of crystallinity of the hydrothermally treated samples was estimated by fitting of the diffraction patterns and was taken as the integrated intensity of the (101) anatase peak divided by the sum of the integrated intensities of the anatase (101) peak and the amorphous feature centred around 29° 2*θ* or brookite (211) peak, as appropriate (Fig. 3(c)). Treatment at 100 °C causes a gradual increase in crystallinity, reaching 74% after 3 hours, increasing to full crystallinity at 17 and 24 hours. At higher treatment temperatures of 150 and 200 °C full crystallinity is attained after just 3 hours. Crystallite sizes were also determined by use of the Scherrer equation applied to the (101) anatase peak (Fig. 3(e)). Similar trends are observed in the calculated anatase crystallite sizes, as the sample treated at 100 °C shows a relatively slow growth, while treatment at 150 °C yields crystallites with sizes of 9–11 nm after 6 or more hours, and 200 °C treatment gives crystallite sizes of 11–13 nm for all treatment times. It is worth noting that crystal growth is seemingly significantly more rapid in the fully crystallised samples (Fig. 3(g)).

The X-ray powder diffraction patterns of the solvothermally treated dispersible titania samples are shown in Fig. 1(d)–(f). These patterns also show a transformation from the amorphous hydrous titania precursor to nanocrystalline anatase phase titania. Unlike the hydrothermally treated samples, however, those treated solvothermally in TFA do not exhibit the diffuse scattering feature at 29° 2*θ*, but rather show a broad feature centred at 21° 2*θ*. This is most likely due to the formation of organo-polytitanate species, which are known to show diffraction features in this region,^47^ or lepidocrocite type structures from further protonation of the amorphous titania structure.^45,46^ The degree of crystallinity of the solvothermally treated samples was estimated by a similar method and was taken as the integrated intensity of the (101) anatase peak divided by the sum of the integrated intensities of the anatase (101) peak and the amorphous feature centred around 21° 2*θ* (Fig. 3(d)). On increasing solvothermal treatment time the broad amorphous contribution gradually decreases in intensity as the anatase phase crystallises. At the lowest reaction temperature of 100 °C the amorphous features persist even up to 24 hours of treatment, with a maximum crystallinity of 44% achieved (it should also be noted fitting of the data for the 3 and 6 hour samples was poor due to the large contribution of the amorphous scattering overlapping with the anatase (101) peak). The higher reaction temperatures of 150 and 200 °C yield fully crystalline phases after 17 and 6 hours, respectively, with traces of the brookite phase. Interestingly the crystallite size of these samples is significantly smaller than those treated hydrothermally, and does not get above a maximum of 4.5 nm until full crystallisation has occurred in the 200 °C treated samples, whereupon increased growth is observed (Fig. 3(f) and (h)).

The unit cell parameters determined from the fitted peak positions of the anatase (101) and (200) peaks are broadly in line with expected values (Fig. 3(a), (b) and Table 1). Bulk anatase exhibits cell parameters of *a* = 3.7842 Å, *c* = 9.5146 Å, *V* = 136.2507 Å^3^ (ICSD 9852). Significant deviations from these values have been observed in numerous instances, particularly associated with extremely small nanoparticles. In many systems small crystallite sizes are associated with an increase in unit cell volume, typically attributed to surface strain.^48–50^ In the case of anatase, similar lattice expansions have also been reported; however anisotropic decreases in unit cell parameters have also been reported.^51^ Here, in the case of the hydrothermally treated undoped samples, the *c* parameter is lower than the bulk in all cases with a minimum of 9.116 Å for the sample prepared at 100 °C for 3 hours, though given the extremely small size and presence of the amorphous phase there is likely a large strain contribution. In general, the *c* parameters increase towards that of the bulk with increasing crystallite size, as may be expected. The *a* parameters of samples prepared at 150 °C and 200 °C are close to the expected value, but samples prepared at 100 °C are significantly larger regardless of treatment time. This behaviour is quite consistent with prior reports on the effects of particle size on anatase unit cell parameters.^51–53^ In contrast the undoped samples treated solvothermally in trifluoroacetic acid show very consistent *a* parameters, in line with the expected bulk values, while the *c* parameters vary significantly, and are generally lower than the bulk value, though they remain within the range of reported values. Notably samples prepared at 150 °C are consistently found to have unit cell parameters closest to those of bulk anatase.

**Table 1:** Properties of undoped titania samples treated hydrothermally and solvothermally in trifluoroacetic acid at varying temperatures and times determined by X-ray diffraction

Temperature (°C)	Time (hours)	Hydrothermal					Solvothermal				
		Unit cell parameters			Crystallinity (%)	Size (nm)	Unit cell parameters			Crystallinity (%)	Size (nm)
		*a* (Å)	*c* (Å)	*V* (Å^3^)			*a* (Å)	*c* (Å)	*V* (Å^3^)		
100	0	—	—	—	0	—	—	—	—	0	—
	3	3.861	9.116	135.9	74	3.6	—	—	—	31	2.0
	6	3.850	9.380	139.0	80	4.1	3.787	9.309	133.5	35	2.6
	17	3.846	9.406	139.1	99	7.3	3.777	9.476	135.2	38	3.0
	24	3.843	9.449	139.5	100	8.1	3.791	9.416	135.3	44	3.2
150	0	—	—	—	0	—	—	—	—	0	—
	3	3.793	9.241	132.9	100	5.5	3.799	9.540	137.7	56	3.0
	6	3.787	9.347	134.1	100	9.1	3.796	9.552	137.6	63	3.8
	17	3.786	9.365	134.3	100	11.1	3.791	9.511	136.7	94	4.6
	24	3.788	9.361	134.3	100	8.9	3.793	9.497	136.6	97	4.5
200	0	—	—	—	0	—	—	—	—	0	—
	3	3.780	9.207	131.6	100	11.3	3.789	9.468	135.9	65	4.3
	6	3.783	9.355	133.9	100	12.6	3.783	9.351	133.9	98	5.8
	17	3.784	9.401	134.6	100	13.7	3.784	9.399	134.6	98	6.3
	24	3.781	9.295	132.9	100	11.6	3.782	9.295	132.9	100	7.7

**Fig. 2:**
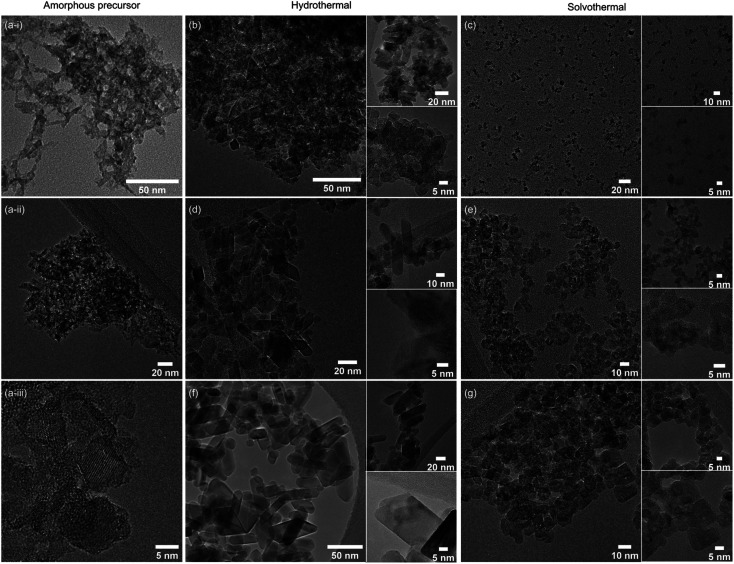
TEM images of undoped amorphous titania precursor (a(i–iii)), and undoped titania treated for 24 hours hydrothermally and solvothermally, respectively, at 100 °C (b and c), 150 °C (d and e), and 200 °C (f and g). Insets show the corresponding higher magnification images and HRTEM where possible.

Further support for the crystallinity and crystallite sizes reported from XRD is provided by transmission electron microscopy (TEM), which was carried out on the hydrous titania precursor and the six undoped anatase samples prepared hydrothermally and solvothermally at 100, 150, and 200 °C for 24 hours, as shown in Fig. 2. While predominantly amorphous under the beam, the final image presented in Fig. 2(a(iii)) provides some evidence for the hypothesis that the precursor gel contains small nano-clusters, which crystallise further by hydro- or solvothermal treatment. Qualitative analysis of the TEM images also confirms the high level of crystallinity obtained hydrothermally across all reaction temperatures in comparison to the solvothermal route, with well-defined decahedral and rod-like prismatic morphologies obtained at 200 °C. These morphologies are typical of the tetragonal crystal system of anatase, and are commonly encountered under hydrothermal synthesis conditions.^54–60^ TEM images of the samples treated solvothermally in trifluoroacetic acid similarly support the XRD analysis. The sample obtained after treatment for 24 hours at 100 °C shows well-dispersed sub-5 nm particles with no obvious lattice fringes, indicating relatively low crystallinity. Samples obtained at the higher temperature of 150 °C show particles with diameters of ∼5 nm. While the particle morphology is largely irregular, a number of these particles broadly exhibit the tetragonal bipyramidal morphology of anatase and lattice fringes are readily observed. After treatment at 200 °C in trifluoroacetic acid the sample consists of irregularly shaped particles with sizes varying between approximately 5 to 10 nm. The crystalline nature of these particles is again readily observed.

**Fig. 3:**
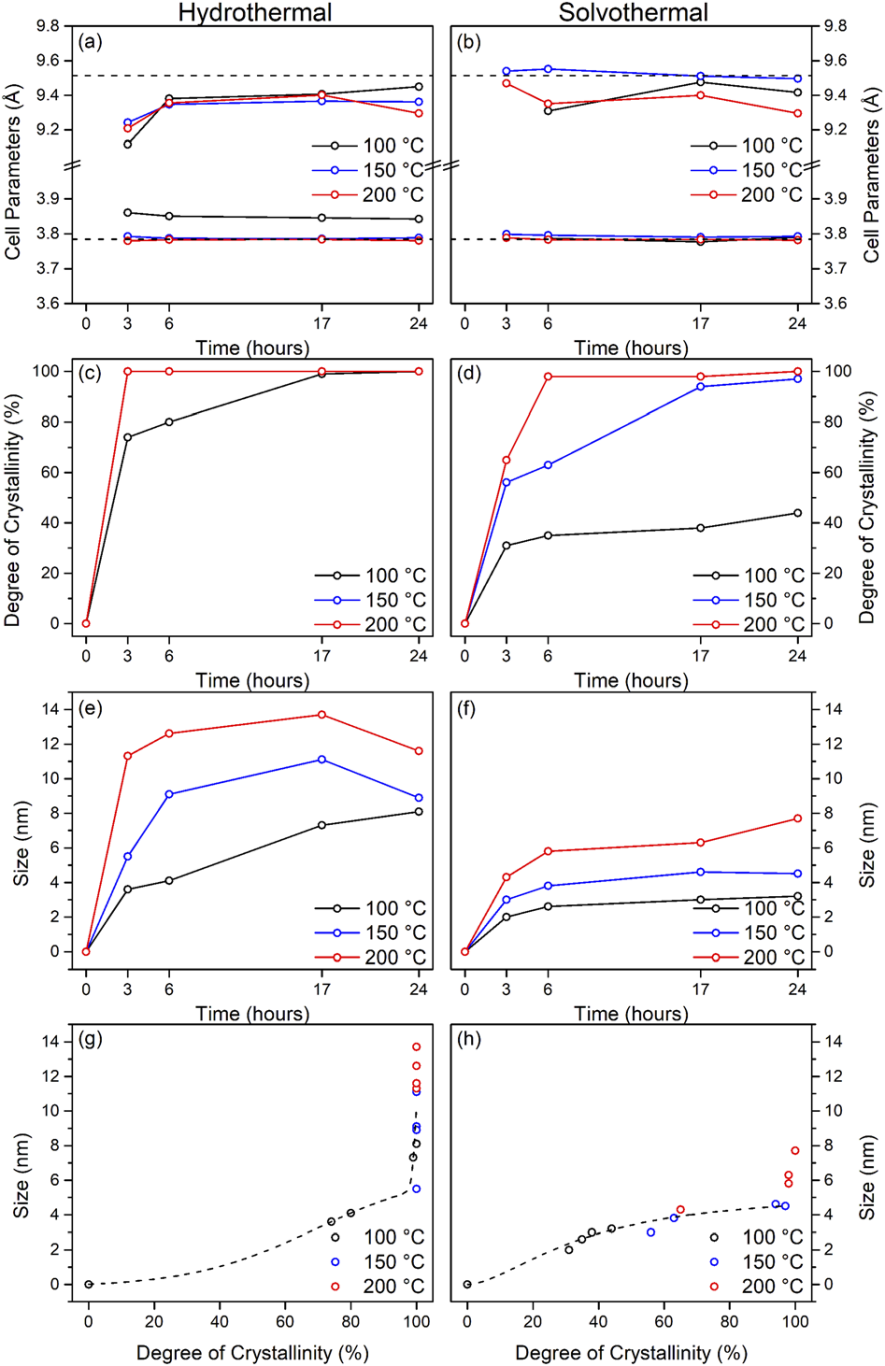
Properties of undoped titania samples treated hydrothermally and solvothermally in trifluoroacetic acid at varying temperatures and times determined by X-ray diffraction. Unit cell parameters of (a) hydrothermally and (b) solvothermally prepared samples, corresponding degree of crystallinity (c and d), crystal sizes (e and f), and size related to crystallinity (g and h). Dashed lines in (a) and (b) show the bulk anatase cell parameters (*a* = 3.7842 Å, *c* = 9.5146 Å, V = 136.2507 Å^3^), those in (g) and (h) highlight the trends relating size and crystallinity for hydrothermal and solvothermal samples, respectively.

Taken as a whole, this suggests that the amorphous precursor gel consists of approximately 4.5 nm domains or clusters, which once fully crystallised by either hydrothermal or solvothermal treatment may undergo more rapid growth by interparticle fusion. Surface capping by trifluoroacetate slows the crystallisation process leading to the apparent plateauing of crystallite size at ∼4.5 nm during solvothermal treatment at intermediate temperatures and times. The properties of the hydrothermally and solvothermally treated undoped samples obtained by XRD are shown in Fig. 3, and summarised in Table 1.

**Fig. 4:**
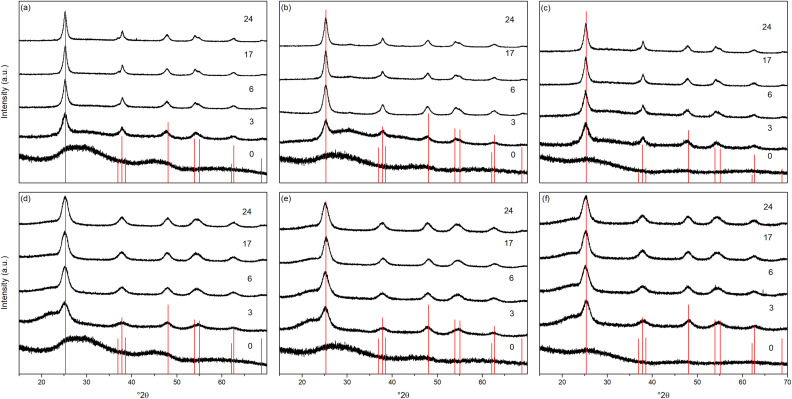
X-ray diffraction patterns of titania doped with (a) nickel, (b) copper, and (c) cobalt treated hydrothermally at 150 °C for the indicated times and the corresponding (d) nickel, (e) copper, and (f) cobalt doped titania treated solvothermally in trifluoroacetic acid at 150 °C for the indicated times. Data have been normalised and offset for clarity.

**Fig. 5:**
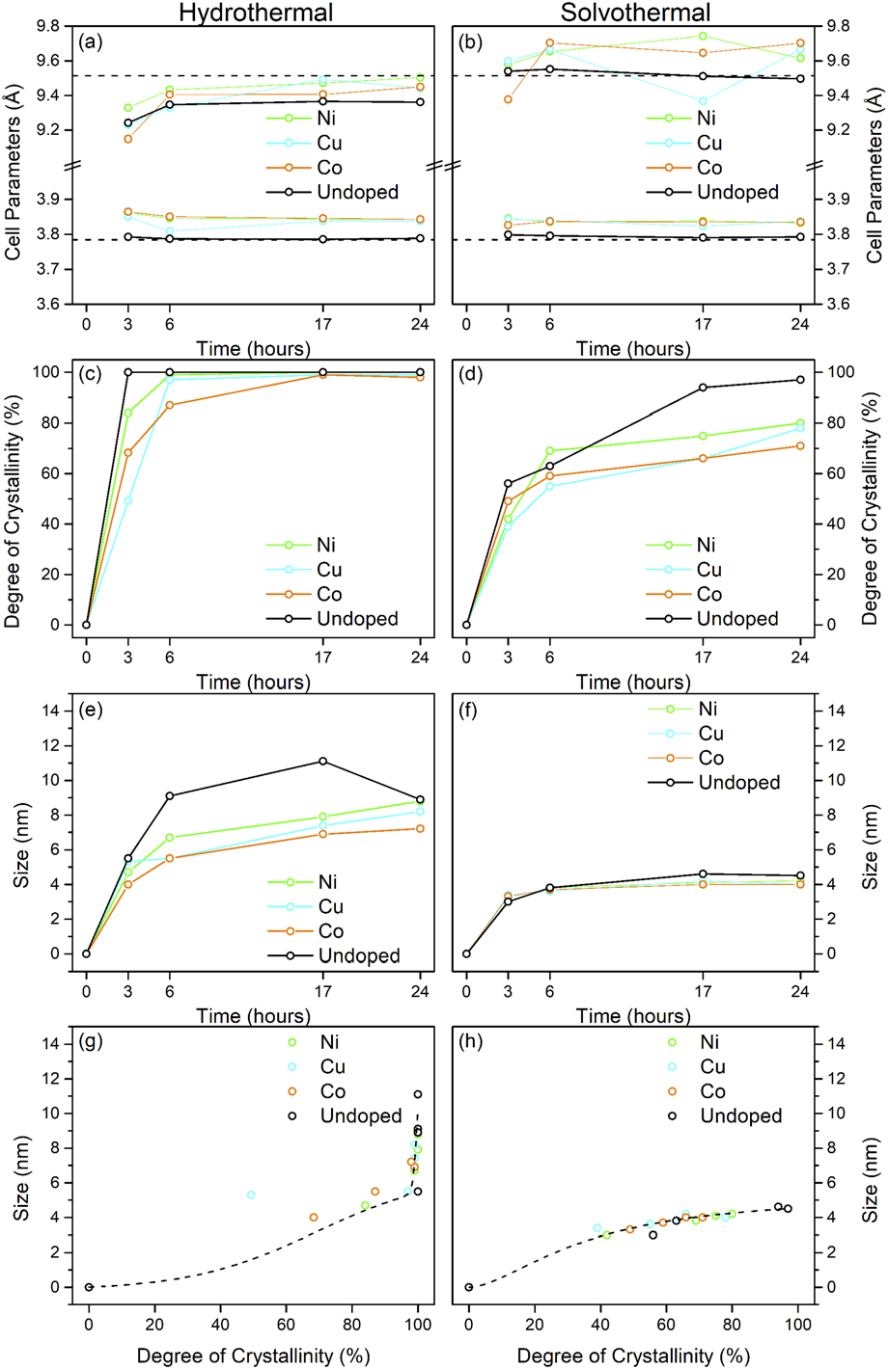
Properties of nickel, copper, and cobalt doped titania samples treated hydrothermally and solvothermally in trifluoroacetic acid at 150 °C and varied times determined by X-ray diffraction. Unit cell parameters of (a) hydrothermally and (b) solvothermally prepared doped samples, corresponding degree of crystallinity (c and d), crystal sizes (e and f), and size related to crystallinity (g and h). The data for the corresponding undoped samples treated at 150 °C are also included in (a–h). Dashed lines in (a) and (b) show the bulk anatase cell parameters (*a* = 3.7842 Å, *c* = 9.5146 Å, *V* = 136.2507 Å^3^), those in (g) and (h) highlight the trends relating size and crystallinity for hydrothermally and solvothermally treated undoped samples, respectively.

The X-ray powder diffraction patterns of the transition metal doped samples treated hydrothermally and solvothermally at 150 °C for varying times are shown in Fig. 4(a–c) and (d–f), respectively. EDX analysis of doped samples treated solvothermally for 24 hours show dopant levels (atomic%) of 4.64 ± 0.10% (Ni), 4.81 ± 0.20% (Cu), and 5.05 ± 0.48% (Co), in line with the target composition of M : Ti of 5 : 95. The doped samples under hydrothermal conditions are slower to achieve full crystallinity, and exhibit smaller crystallite sizes than their undoped counterparts (Fig. 5 and Table 2). Similarly, under solvothermal conditions, the transition metal doped samples are significantly less crystalline, only attaining a maximum crystallinity of 80–88% after 24 hours, in contrast to the undoped analogue. This suggests that the presence of the dopants limits the crystallisation of the anatase phase, which is consistent with previous reports on the crystallisation and growth of doped titanium dioxide.^61^ All doped samples show broader peaks in comparison to their undoped counterparts, again suggesting that the presence of the dopants hinders anatase growth leading to smaller crystallite sizes, though it should be noted that the peak broadening may also result from strain or disorder induced by the presence of the dopants within the structure. Williamson–Hall analysis of selected samples indicates that some degree of strain is indeed present in both undoped and doped samples, however significant overlapping of peaks (especially those around 38° 2*θ*) limits the reliability of this approach for quantifying these effects. The unit cell parameters of the transition metal doped samples are similarly in line with expected values, and in almost all cases, both hydrothermally and solvothermally treated doped samples show a significant increase in cell volume in comparison to their undoped counterparts (Fig. 5(a), (b) and Table 2). This is a strong indication that the dopant ions, all of which have larger ionic radii than Ti^4+^, have incorporated into the anatase structure.^62–65^ Given the extremely small sizes of the obtained particles and the known impact of particle size on cell parameters, however, more detailed conclusions on the nature of the doping cannot be drawn. The properties of the hydrothermally and solvothermally treated doped samples derived from analysis of their XRD patterns are shown in Fig. 5, and are summarised in Table 2.

**Table 2:** Properties of doped titania samples treated at 150 °C hydrothermally and solvothermally in trifluoroacetic acid determined by X-ray diffraction

Dopant	Time (hours)	Hydrothermal					Solvothermal				
		Unit cell parameters			Crystallinity (%)	Size (nm)	Unit cell parameters			Crystallinity (%)	Size (nm)
		*a* (Å)	*c* (Å)	*V* (Å^3^)			*a* (Å)	*c* (Å)	*V* (Å^3^)		
Nickel	0	—	—	—	0	—				0	—
	3	3.861	9.329	139.1	84	4.7	3.846	9.580	141.7	42	3.0
	6	3.846	9.433	139.5	99	6.7	3.836	9.654	142.1	69	3.8
	17	3.844	9.472	139.9	100	7.9	3.838	9.744	143.5	75	4.1
	24	3.843	9.503	140.4	100	8.8	3.833	9.616	141.3	80	4.2
Copper	0	—	—	—	0	—	—	—	—	0	—
	3	3.850	9.229	136.8	49	5.3	3.844	9.600	141.9	39	3.4
	6	3.809	9.330	135.4	97	5.5	3.839	9.664	142.4	55	3.6
	17	3.838	9.493	139.8	99	7.4	3.824	9.368	137.0	66	4.2
	24	3.838	9.443	139.1	99	8.2	3.837	9.666	142.3	78	4.0
Cobalt	0	—	—	—	0	—	—	—	—	0	—
	3	3.865	9.149	136.7	67	4	3.826	9.376	137.3	49	3.3
	6	3.851	9.405	139.5	87	5.5	3.837	9.705	142.9	59	3.7
	17	3.846	9.406	139.1	99	6.9	3.835	9.646	141.9	66	4.0
	24	3.843	9.449	139.6	98	7.2	3.835	9.704	142.7	71	4.0

**Fig. 6:**
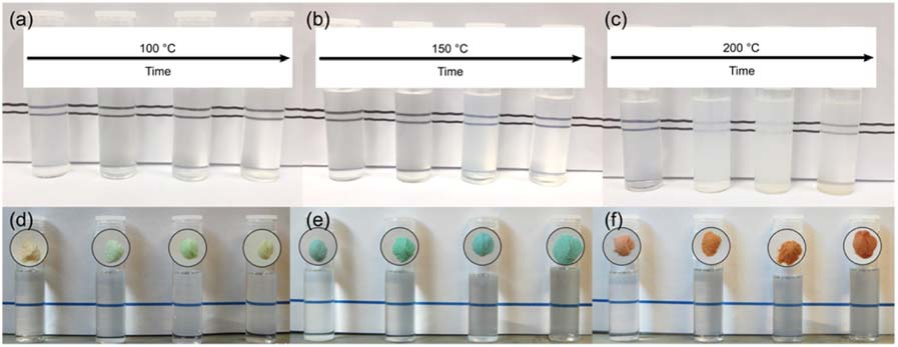
Photographs of 0.1 g of the surface modified samples dispersed in 10 mL of acetone. Undoped samples obtained after treatment in trifluoracetic acid at 100 °C (a), 150 °C (b), and 200 °C (c). Nickel (d), copper (e), and cobalt (f) doped samples obtained after treatment in trifluoracetic acid at 150 °C are shown alongside their corresponding isolated powders. Treatment times (3, 6, 17, and 24 hours) increase from left-to-right in each case.

As shown in Fig. 6(a)–(c) all undoped solvothermally treated particles are readily dispersible in acetone, yielding transparent solutions, with the exception of those particles which are both fully crystalline, and have an average crystallite size ≥4.5 nm (*i.e.* those obtained after solvothermal treatment at 150 °C for 17 and 24 hours, and 200 °C for 6 hours or more). Achieving these transparent solutions does not require any additional sonication, surfactants, or thermal treatment. Even in the case of the larger particles obtained by solvothermal treatment at 150 °C for 17 and 24 hours and 200 °C for 6–17 hours stable, light scattering, colloidal dispersions are obtained. Only the largest 7.7 nm particles obtained after 24 hours of treatment at 200 °C do not form stable dispersions. All doped samples prepared by solvothermal treatment in trifluoroacetic acid at 150 °C also give fully transparent stable solutions in acetone (Fig. 6(d)–(f)).

We have previously ascribed the apparent solubility of trifluoroacetate modified tin and titanium oxide nanoparticles to the interaction between the polar –CF_3_ group of relatively strongly bound bidentate trifluoroacetate with the ketone moiety of acetone or polar groups of similarly polar aprotic solvents.^38,39^

**Fig. 7:**
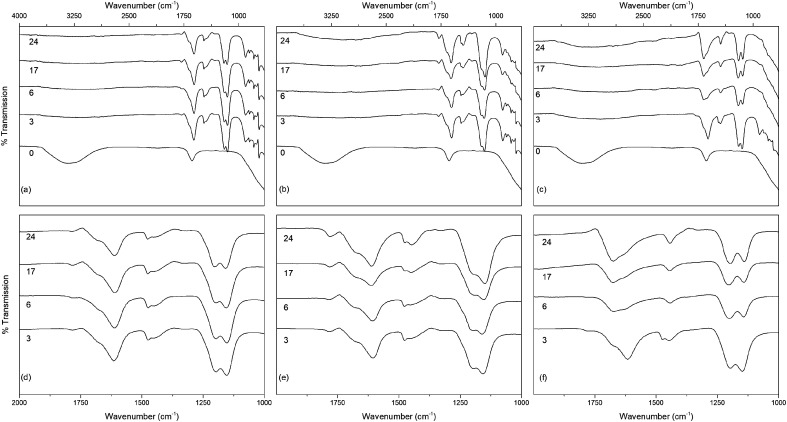
The infrared spectra of undoped titania treated solvothermally in trifluoroacetic acid for the indicated times at (a) 100 °C, (b) 150 °C and (c) 200 °C, with the corresponding carboxylate stretching regions highlighted in (d–f). Data have been normalised and offset for clarity.

**Fig. 8:**
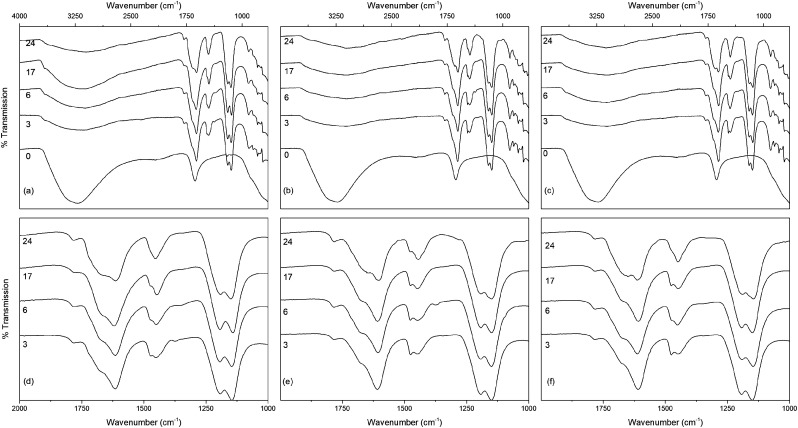
The infrared spectra of titania treated solvothermally in trifluoroacetic acid for the indicated times at 150 °C doped with (a) nickel, (b) copper, and (c) cobalt, with the corresponding carboxylate stretching regions highlighted in (d–f). Data have been normalised and offset for clarity.

The infrared spectra of the solvothermally treated undoped samples are shown in Fig. 7. As would be expected the amorphous hydrous precursor shows only those bands associated with surface water and hydroxyl groups at 1640 cm^−1^ and 3300 cm^−1^. Upon solvothermal treatment in trifluoroacetic acid these are replaced by bands corresponding to coordinated trifluoroacetate groups. The spectra of the trifluoroacetate modified anatase nanocrystals treated at the lower temperature of 100 °C exhibit weak bands at 1785 cm^−1^, indicative of physisorbed or hydrogen bonded trifluoroacetate, as well as broad envelopes at 1616 cm^−1^ with a shoulder at 1681 cm^−1^ which can be ascribed to the asymmetric stretch of coordinated carboxylate (*ν*_as_(OCO)), coupled with symmetric stretching (*ν*_s_(OCO)) bands at 1476 cm^−1^ and 1446 cm^−1^. Strong bands are observed at 1147 cm^−1^ and 1199 cm^−1^, attributed to *ν*_as_(CF_3_) and *ν*_s_(CF_3_) + *ν*_s_(OCO), respectively. Additional characteristic trifluoroacetate bands at 911 cm^−1^, 850 cm^−1^, and 791 cm^−1^ also appear, while a very shallow envelope remains in the hydroxyl stretching region between 3700 cm^−1^ and 2000 cm^−1^.^38,39,66–68^ Varying reaction time does not cause any significant changes to the spectra of these samples, however at higher reaction temperatures significant variations are observed in the carboxylate stretching region between 1800 cm^−1^ and 1300 cm^−1^. The separation of *ν*_as_(OCO) and *ν*_s_(OCO) bands, given as Δ*ν*, is well known as an indicator of the mode of carboxylate coordination. In the case of trifluoroacetate coordination complexes Δ*ν* values of approximately 150 cm^−1^ or less are typically associated with bidentate coordination, while larger values are indicative of asymmetric bridging bidentate or unidentate coordination. This region for all undoped solvothermally synthesised samples is highlighted in Fig. 7(d)–(f), and clear trends can be seen at higher reaction temperatures and times. The band at 1616 cm^−1^ decreases in intensity, as does the band at 1476 cm^−1^. At the same time the shoulder at 1681 cm^−1^ becomes a more pronounced band, and the relative intensity of the band at 1446 cm^−1^ also increases. These changes represent a shift from bidentate coordination with a Δ*ν* value of ∼140 cm^−1^ towards unidentate coordination with Δ*ν* values above 200 cm^−1^ becoming dominant. There is also an apparent increase in intensity of the band at 1200 cm^−1^ with increasing reaction time, which may also indicate increasing unidentate character. These trends are replicated across the doped samples as well (shown in Fig. 8), suggesting that the shift from bidentate to unidentate coordination is largely unaffected by the presence of the transition metal dopants. It is noteworthy that those samples which yield the poorest dispersions are those obtained after treatment at 200 °C for 6 to 24 hours, consistent with the least bidentate character by infrared. This may indicate that the trifluoroacetate moieties associated with these particles are only weakly bound.

**Fig. 9:**
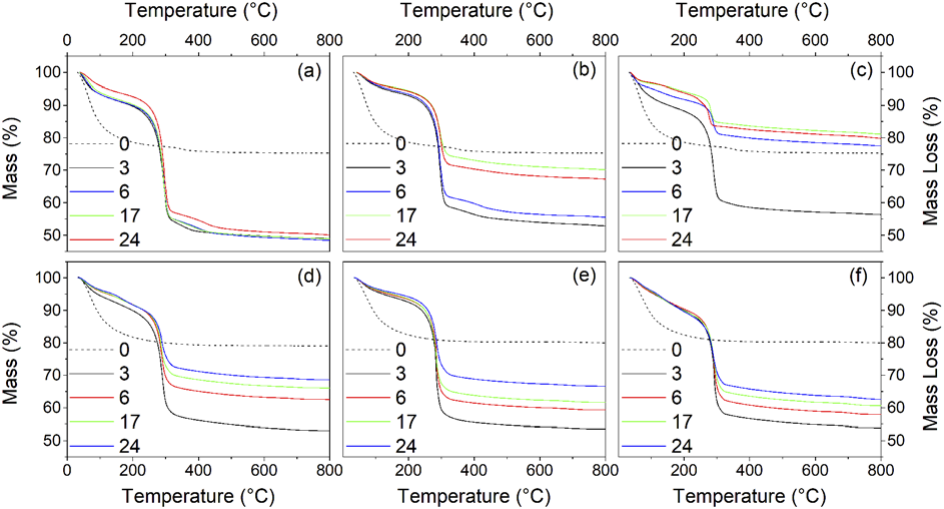
Thermogravimetry traces of the undoped surface modified titania samples obtained after treatment in trifluoracetic acid at 100 °C (a), 150 °C (b), 200 °C (c), and nickel (d), copper (e), and cobalt (f) doped samples obtained after treatment in trifluoracetic acid at 150 °C for the indicated times (hours). Dashed lines show the TG traces of the corresponding amorphous precursors.

Thermogravimetry (TG) traces of the undoped and doped solvothermally treated products are shown in Fig. 9. Thermal analysis of the hydrous precursors shows a single smooth weight loss step of ∼20% up to 400 °C in all cases. In contrast the TG traces of the dispersible trifluoroacetate modified anatase nanocrystals show several distinct steps. All solvothermally treated samples show a slight weight loss up to 90 °C attributed to the loss of loosely bound physisorbed or hydrogen bonded trifluoroacetic acid. This is followed by a shallower weight loss step up to 250 °C, at which point the remaining bound trifluoroacetate undergoes rapid decomposition. The nickel and cobalt doped samples deviate slightly from this pattern with very minor additional weight loss steps apparent at 150 °C and 120 °C, respectively, the origins of which are unclear, but may be due to the dehydration of trace amounts of the corresponding metal hydroxides or the loss of more strongly physisorbed trifluoroacetic acid.

**Fig. 10:**
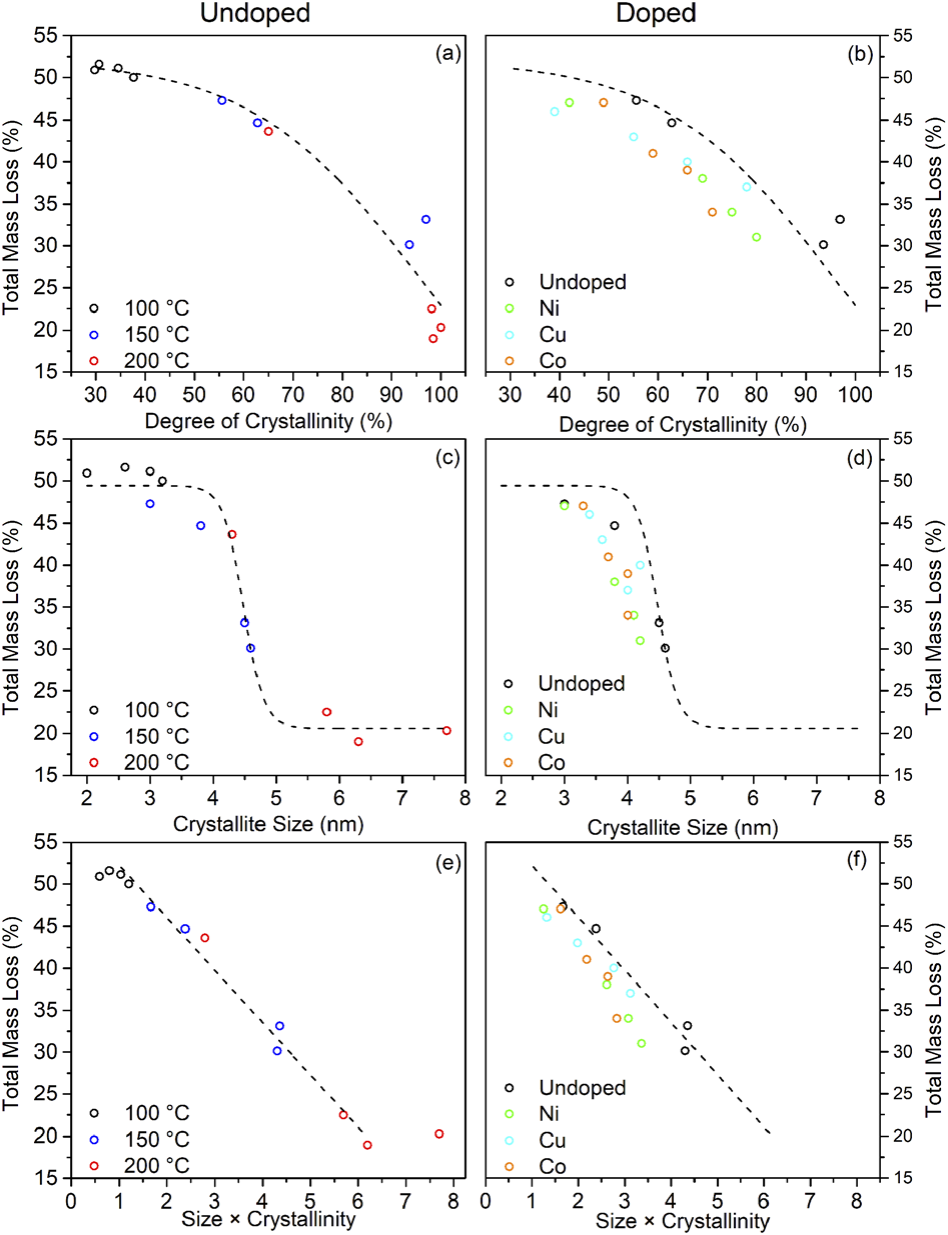
The relationship between the total mass loss by TGA and the degree of crystallinity (a and b), crystallite size (c and d), and their combination (e and f) for undoped and doped surface modified samples. Dashed lines highlight the trends observed for the undoped samples.

What is clear from the TG traces is that increasing the solvothermal reaction temperatures and times leads to a decrease in the quantity of bound trifluoroacetate. The decreasing total mass loss of each sample, adjusted to disregard the contribution of physisorbed species lost below 100 °C, shows a direct correlation with increasing crystallinity and size determined by XRD. In particular it can be seen that the decrease in trifluoroacetate content is relatively smooth with respect to increasing crystallinity, but drops sharply as the anatase crystallites attain a size of 4.5 nm. In conjunction with the larger crystallite sizes observed in the solvothermally treated samples after full crystallisation has been achieved, this would suggest that crystallisation is initially an internal rearrangement of amorphous particles of approximately 4.5 nm diameter with the conversion of trifluoroacetate from bidentate to unidentate coordination, as shown by the IR spectra, and eventual loss of bound trifluoroacetate groups during Ti–O–Ti bond formation. Once full crystallisation of these cores is achieved the particles may subsequently undergo fusion growth of the crystallites, as shown by the large sizes attained in the 200 °C treated samples. Doped samples prepared at 150 °C show similar behaviour, albeit with lower crystallinity and smaller crystallite sizes in comparison to their undoped analogues. These trends are highlighted in Fig. 10(a)–(d).

The combination of crystallisation and growth leads to an almost linear relationship with the trifluoroacetate content, as highlighted in Fig. 10(e) and (f). Interestingly, attributing the total mass loss purely to trifluoroacetate (CF_3_COO^−^) and extrapolating the linear portion of this data back to a hypothetical 0% crystallinity, zero size particle gives a nominal mass loss of 58.2%. This corresponds to an empirical formula of TiO(OH)(CF_3_COO), analogous to many titanium(iv) oxo carboxylate clusters; clusters which invariably involve bridging bidentate carboxylate groups.^69–72^

Taking the fully crystalline trifluoroacetate modified anatase samples prepared by solvothermal treatment at 150 °C for 24 hours and at 200 °C for 6 or more hours, it is possible to estimate the degree of surface coverage achieved by this system. Based on the 150 °C sample the average crystallite diameters calculated from the XRD pattern of 4.5 nm gives the volume and surface area of the “average” modified anatase nanoparticle as 48 nm^3^ and 64 nm^2^, respectively. The total mass loss of bound trifluoroacetate of 31% (corrected for loosely bound physisorbed species) corresponds to 489 molecules of trifluoroacetate per particle. This gives a surface coverage of 6.9 molecules of trifluoroacetate per nm^2^. Similar calculations may be performed for the larger fully crystallised samples from the 200 °C reactions yielding the surface coverage of 5–7 CF_3_COO^−^ nm^−2^, as shown in Table 3.

**Table 3:** Surface coverage of fully crystalline trifluoroacetate modified anatase nanocrystals

Temperature (°C)	Time (hours)	Crystallinity (%)	Size (nm)	Volume (nm^3^)	Surface area (nm^2^)	Total mass loss (%)	CF_3_COO^−^:TiO_2_	CF_3_COO^−^/nm^2^	Coverage^a^ (%)
								Molecules	
150	24	97	4.5	48	64	31	0.31	6.9	111–149
200	6	98	5.8	102	106	22	0.20	5.8	84–111
200	17	98	6.3	131	124	19	0.17	5.1	74–98
200	24	100	7.7	239	186	20	0.18	6.8	98–130

a Available surface Ti atoms taken as 5.2 nm^−2^ (101) to 6.9 nm^−2^ (001).

Typically, the exposed facets of sol–gel derived and hydrothermally prepared anatase nanoparticles are dominated by the (101) and (001) faces, with the (101) face generally most highly expressed.^56–58,73,74^ Computational studies have shown that the surface density of unsaturated Ti sites on these faces are 5.2 nm^−2^ and 6.9 nm^−2^.^75,76^ Given the general dominance of the (101) face in anatase nanoparticles, this would suggest that this solvothermal crystallisation of anatase from amorphous titania in trifluoroacetic acid generally results in near total or excess coverage of the obtained nanoparticles.

**Scheme 1:**
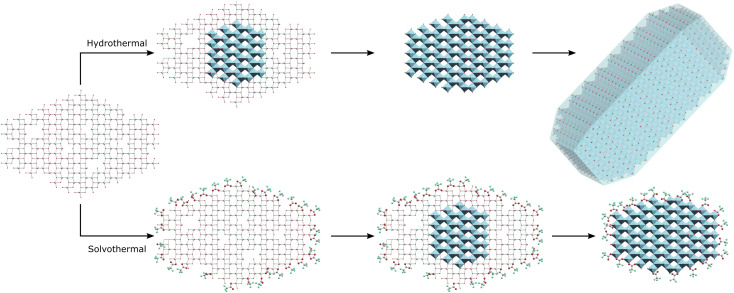
Hydrothermal treatment of amorphous titania leads to intraparticle crystallisation of the anatase phase and subsequent growth by interparticle fusion, while solvothermal treatment in trifluoroacetic acid initially leads to complete surface modification with intraparticle crystallisation yielding surface capped anatase phase hybrid nanocrystals.

Based on these results it may be suggested that amorphous gel particles with domain sizes of ∼4.5 nm are formed by precipitation of titania from TiCl_4_ at pH 6. Hydrothermal treatment of this precursor allows rapid internal rearrangement to the anatase phase and subsequent fast growth by particle fusion. Solvothermal treatment in TFA leads to a change in the internal structure of the gel towards an amorphous organo-titanate or lepidocrocite type species, with bonding of trifluoroacetate to every available Ti site, mostly through a bridging bidentate arrangement similar to that found in titanium(iv) oxo carboxylate clusters. These carboxylate modified gel particles are slower to undergo internal rearrangement to the anatase phase, as there must also be a decrease in binding of the trifluoroacetate groups to the titanium ions, as evidenced by a shift from bidentate to unidentate bonding on crystallisation. Once full crystallisation has been achieved rapid growth by particle fusion may occur again. It is important to note that all available Ti sites are coordinated by trifluoroacetate, resulting in complete surface coverage. It is thus suggested that intraparticle crystallisation, which it seems happens quite readily as even 100 °C is sufficient for full crystallisation under hydrothermal conditions, occurs first, followed by subsequent interparticle fusion and growth once the anatase phase extends throughout the particle. It is possible that interparticle fusion may be happening simultaneously; however, the apparent size limit of ∼4–5 nm is observed in both hydrothermal and solvothermal cases and is seemingly only overcome when full crystallinity is achieved, indicating that crystallisation and growth occur in a step-wise fashion. This interparticle growth process is also quite hindered by the use of trifluoroacetic acid as it forms an effective barrier to interparticle fusion, hence the solvothermally treated particles retain their small 4–5 nm size unless treated at higher temperatures of 200 °C for extended times – sufficient to cause a significant change in the nature and quantity of bound trifluoroacetate as shown by IR spectra and thermal analysis. This suggested mechanism is highlighted in Scheme 1. This high degree of surface coverage with trifluoroacetate leads to those particles which have not undergone significant growth (crystallite size < 5 nm) being highly dispersible in acetone.

The solubility or dispersibility of the obtained nanoparticles can be attributed to a combination of factors, primarily size and surface modification. All but the largest, most crystalline particles with the least amount of more strongly bound bidentate trifluoroacetate yield stable dispersions. In the case of those particles which do not exhibit full crystallinity, transparent solutions are obtained in acetone. The ease of dispersion and the transparency of the resulting solutions can be attributed to a non-crystalline titanium trifluoroacetate interface between the anatase phase cores and the solvent, such that interaction of this interface with the solvent leads to a gradation of refractive indices and sufficiently strong solvation to solubilise the hybrid anatase nanoparticles.

The formation of transition metal doped analogues is readily achieved by incorporation of dopant ions into the initially formed amorphous precursor. Doping in this way does not seem to interfere significantly with any of the behaviour above, beyond appearing to disrupt or delay, but not fully prohibit, the crystallisation of the anatase phase.

## Conclusions

Solvothermal treatment of amorphous titania in trifluoroacetic acid yields highly dispersible surface modified anatase phase nanoparticles. The reaction of the amorphous precursor with trifluoroacetic acid led to the crystallisation and growth of anatase phase titania, initially by an internal rearrangement yielding 4.5 nm particles, which undergo further growth at higher temperatures and longer reaction times by interparticle fusion. Comparison with the behaviour of hydrothermally treated analogues indicates that the domain size of 4.5 nm is an inherent feature of the amorphous titania precursor obtained by precipitation from titanium tetrachloride at pH 6. In addition to inducing crystallisation of the anatase phase, the solvothermal treatment in trifluoroacetic acid results in the complete coordination of all available titanium sites by trifluoroacetate leading to total surface coverage of the obtained anatase nanoparticles, with almost all surface titanium ions coordinated by trifluoroacetate groups. This surface modification renders the obtained hybrid nanoparticles highly dispersible in polar aprotic solvents such as acetone. Incorporation of transition metal dopants is readily achieved by coprecipitation of the dopant ions with the amorphous precursor. These doped precursors behave in the same way, with solvothermal treatment yielding dispersible, surface modified, doped anatase nanoparticles.

This approach provides an easy, scalable, and tuneable synthetic route to hybrid anatase phase titanium dioxide nanoparticles, and potentially other hybrid metal oxides. The incorporation of dopant metals does not compromise the particle properties, making this procedure very promising for the many and varied applications in which the use of solution processable nanoparticles with tuneable optoelectronic properties are critical.

## Conflicts of interest

There are no conflicts of interest to declare.

## Supplementary Material
